# Mevalonate kinase deficiencies: from mevalonic aciduria to hyperimmunoglobulinemia D syndrome

**DOI:** 10.1186/1750-1172-1-13

**Published:** 2006-04-26

**Authors:** Dorothea Haas, Georg F Hoffmann

**Affiliations:** 1 Department of General Pediatrics, University Children's Hospital Heidelberg, Im Neuenheimer Feld 150, D-69120 Heidelberg, Germany

## Abstract

Mevalonic aciduria (MVA) and hyperimmunoglobulinemia D syndrome (HIDS) represent the two ends of a clinical spectrum of disease caused by deficiency of mevalonate kinase (MVK), the first committed enzyme of cholesterol biosynthesis. At least 30 patients with MVA and 180 patients with HIDS have been reported worldwide. MVA is characterized by psychomotor retardation, failure to thrive, progressive cerebellar ataxia, dysmorphic features, progressive visual impairment and recurrent febrile crises. The febrile episodes are commonly accompanied by hepatosplenomegaly, lymphadenopathy, abdominal symptoms, arthralgia and skin rashes. Life expectancy is often compromised. In HIDS, only febrile attacks are present, but a subgroup of patients may also develop neurological abnormalities of varying degree such as mental retardation, ataxia, ocular symptoms and epilepsy. A reduced activity of MVK and pathogenic mutations in the *MVK *gene have been demonstrated as the common genetic basis in both disorders. In MVA, the diagnosis is established by detection of highly elevated levels of mevalonic acid excreted in urine. Increased levels of immunoglobulin D (IgD) and, in most patients of immunoglobulin A (IgA), in combination with enhanced excretion of mevalonic acid provide strong evidence for HIDS. The diagnosis is confirmed by low activity of mevalonate kinase or by demonstration of disease-causing mutations. Genetic counseling should be offered to families at risk. There is no established successful treatment for MVA. Simvastatin, an inhibitor of HMG-CoA reductase, and anakinra have been shown to have beneficial effect in HIDS.

## Disease name and synonyms

Mevalonic aciduria (MVA, OMIM 251170).

Hyperimmunoglobulinemia D and periodic fever syndrome; Periodic fever, Dutch type (HIDS, OMIM 260920).

## Definition and diagnostic criteria

**Figure 1:**
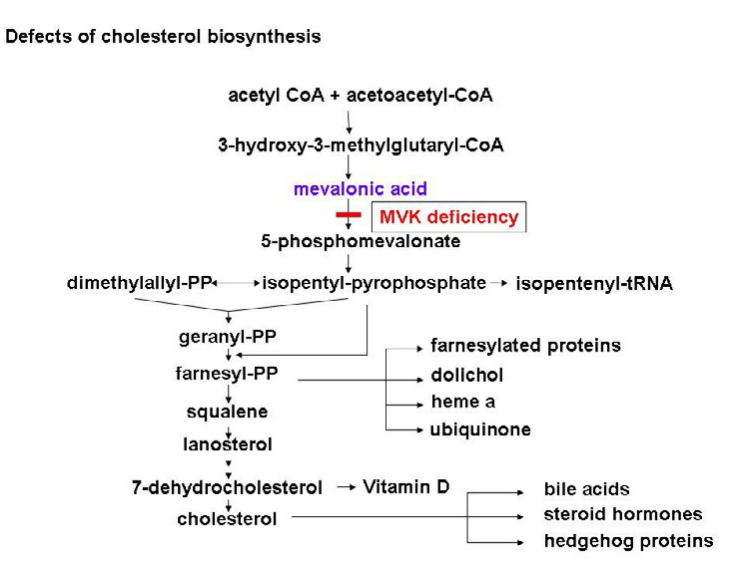
Pathway of cholesterol biosynthesis, showing the defect in mevalonate kinase (MVK) deficiency.

MVA is an autosomal recessively inherited disorder caused by deficiency of mevalonate kinase (MVK; E.C. 2.7.1.36; ATP:(R)-mevalonate 5-phosphotransferase) and identified as the first defect in cholesterol biosynthesis (Figure 1) by Hoffmann *et al*. in 1986 [1]. Mutations in the *MVK *gene and reduced activity of MVK have been identified as underlying cause of both MVA and HIDS syndrome.

MVA is caused by homozygosity or compound heterozygosity for disease-causing mutations in the *MVK *gene, which has been localized to chromosome 12q24 [2]. MVA is biochemically characterized by accumulation of mevalonic acid and mevalonolactone.

The diagnosis of MVA should be suspected in patients with mild dysmorphic features, progressive cerebellar ataxia, psychomotor retardation, failure to thrive, hepatosplenomegaly and recurrent febrile episodes. Uveitis, retinitis pigmentosa and cataracts, as well as myopathy may develop in childhood and adolescence.

HIDS is clinically characterized by recurrent fever episodes starting in infancy and associated with lymphadenopathy, arthralgia, gastrointestinal problems and skin rashes.

A subgroup of HIDS patients may also develop neurological abnormalities of varying degree, such as mental retardation, ataxia, ocular symptoms and epilepsy, a finding that confirms the existence of a continuous spectrum between MVA and HIDS [3].

The diagnosis is established by the detection of elevated excretion of mevalonic acid in urine (MVA) or increased immunoglobulins (Ig) D and A in combination with elevated excretion of mevalonic acid (HIDS). The diagnosis is confirmed by demonstration of deficient MVK enzyme activity or by identification of two disease-causing mutations in the *MVK *gene.

## Differential diagnosis

The constellation of congenital malformations, hepatosplenomegaly, cholestatic liver disease, lymphadenopathy, anemia, severe failure to thrive and developmental retardation, which is found in severely affected MVA patients might suggest chromosomal aberrations or congenital infections. When hematological abnormalities such as anemia, leukocytosis, thrombocytopenia and abnormal blood cell forms predominate, myelodysplastic syndromes may be suspected. Moderately affected MVA patients may be classified among those with psychomotor retardation, myopathy and ataxia.

Recurrent crises from infancy of fever, diarrhea and mucocutaneous manifestations might suggest infectious or autoimmune disease [4]. The development of uveitis in some patients parallels that seen in juvenile rheumatoid arthritis [5]. If developmental delay and neurological symptoms are neither present nor prominent, the differential diagnosis is likely to focus within the group of auto-inflammatory disorders. This group consists of other inherited syndromes: familial Mediterranean fever (FMF); TNF receptor-associated periodic syndrome (TRAPS); familial cold autoinflammatory syndrome/Muckle-Wells syndrome/chronic infantile neurological cutaneous and articular syndrome (FCAS/MWS/CINCA) and periodic fever, aphthous ulcers, pharyngitis, adenitis (PFAPA).

## Epidemiology

MVA is a rare disease. So far, approximately 30 patients have been reported. HIDS seems to be more common. In 2001, the number of reported HIDS patients was about 180 [6] and more patients have been identified since.

## Etiology

For MVA, seven mutations have been identified in 10 out of 20 known patients [7]. Most of these mutations cluster in the C-terminal region of the protein.

Most patients with HIDS are compound heterozygotes for missense mutations in the *MVK *gene. One mutation, V337I, is present in more than 80% of patients [8]. This mutation primarily affects maturation (folding) of the protein resulting in temperature-sensitive expression of MVK *in vivo *[9]. In HIDS patients, MVK may have a residual activity of 5%–15%. In contrast, no residual activity is present in MVA patients [4].

## Clinical description

### Mevalonic aciduria

**Figure 2:**
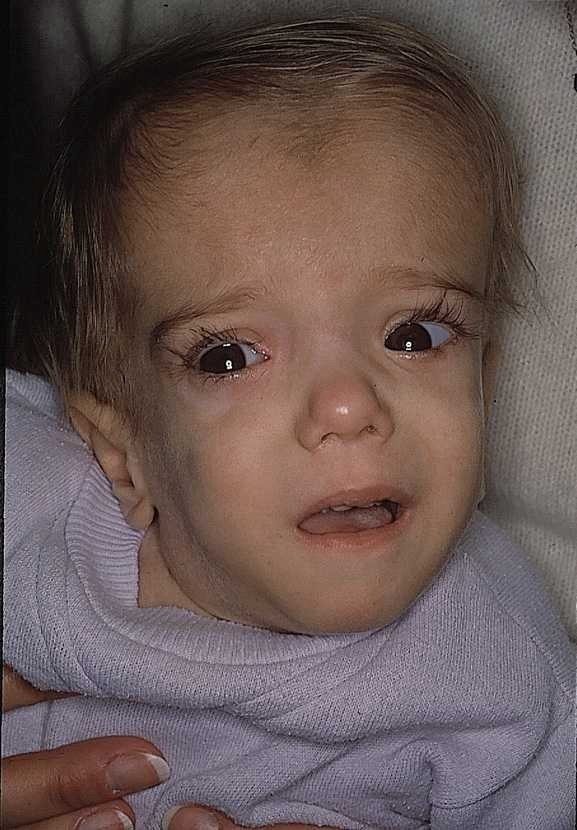
A patient with mevalonic aciduria at the age of 21 months displaying the characteristic facial dysmorphism.

MVA shows considerable clinical heterogeneity. Severely affected patients present from birth with congenital malformations such as microcephaly, dolichocephaly and wide irregular fontanels, as well as low set and posteriorly rotated ears, downslanted palpebral fissures, blue sclerae, and central cataracts (Figure 2). Cholestatic liver disease [10] may be present, and patients may die from recurrent septicemia [11]. Stillbirths with skeletal malformations have been observed in affected families, possibly resulting from the same genetic defect. Cardinal manifestations from late infancy include mild to severe psychomotor retardation, recurrent crises (fever, vomiting and diarrhea), failure to thrive, hypotonia and myopathy. Sometimes hematological abnormalities predominate with normocytic hypoplastic anemia, leukocytosis, thrombocytopenia and abnormal blood cell forms [10], leading to misdiagnoses of congenital infection or myelodysplastic syndromes.

After preschool age, short stature, ataxia due to a progressive cerebellar atrophy and ocular involvement with uveitis, cataracts and tapetoretinal degeneration become predominant findings, and can be the major manifestations in milder cases [3-5,12].

Most patients suffer from frequent crises characterized by fever, vomiting and diarrhea. These episodes appear to be noninfectious in origin, and are often accompanied by arthralgia, subcutaneous edema and a morbilliform rash. Two patients developed uveitis, which worsened during crises. In childhood, episodes occur as often as 25 times per year, lasting in average 4 to 5 days. Laboratory investigations reveal elevated acute phase reactants (erythrocyte sedimentation rate, C-reactive protein, leukocytosis) and elevated immunoglobulins, including IgA, IgE and especially IgD. Anemia, elevations of creatine kinase (CK) and transaminases are present in the more severely affected patients, and worsen during crises. Over the years, the severity and the frequency of the attacks decline.

### Hyperimmunoglobulinemia D syndrome

In HIDS, the clinical picture is dominated by recurrent febrile attacks that usually start before the end of the first year of life [13]. The fever lasts 4 to 6 days and can be provoked by vaccination, minor trauma, surgery or stress. It is usually associated with abdominal pain, vomiting, diarrhea and cervical lymphadenopathy. Other common symptoms include hepatosplenomegaly, headache, arthralgia and rashes. Occasionally, patients present with oral and vaginal aphthous ulcers. After the attack, the patients are free of symptoms, although skin and joint symptoms disappear slowly [6]. Most patients display neither malformations nor neurological abnormalities. However, a subgroup of adult patients also develop neurological abnormalities of varying degree, such as mental retardation, ataxia, ocular symptoms and epilepsy, reflecting the existence of a continuous spectrum between MVA and HIDS [3].

## Diagnostic methods

Generally, the metabolic abnormalities seen in MVA are not those suggestive of an inherited organic aciduria. There is no episodic metabolic decompensation, hypoglycemia, metabolic acidosis, ketosis or hyperammonemia. The key for diagnosis is the elevated level of mevalonic acid in the urine, plasma and cerebrospinal fluid, which can be detected through organic acid analysis [14]. However, in patients with a mild form of MVA and especially in HIDS patients, elevations measured by routine gas chromatography/mass spectrometry may only be found during febrile attacks. The sensitivity of general organic acid analysis is inadequate for recognizing the very low concentrations of mevalonic acid present in control urine. Even moderate, but definitely pathological, elevations can remain below the detection limit [14], and must be verified by isotope dilution mass spectrometry [15]. As the absolute level of mevalonic acid is always elevated in patients with MVK deficiency, samples from patients with a suspected MVA deficiency should undergo a sensitive measurement of mevalonic acid by stable isotope dilution analysis to detect even slight abnormalities. The diagnosis of MVA should be confirmed by mutational analysis or by a radiometric assay of MVK in white blood cells or cultured fibroblasts [16,17]. Urinary excretion of leukotriene E4 is elevated in most patients, with a positive linear correlation with increased mevalonic acid excretion [18].

HIDS is diagnosed on the basis of characteristic clinical findings and continuously high IgD values (more than 100 IU/ml). However, IgD may be normal in patients under three years of age [19]. Consistently normal IgD levels were found in two patients with typical clinical findings and genotype for the syndrome [3,20]. More than 80% of patients have, in addition, high IgA levels [19,21]. During febrile episodes, urinary mevalonate concentrations were found to be significantly elevated in patients with at least one mutation in the *MVK *gene, but slight elevations were also detected between the fever attacks [22]. Therefore, specific sensitive determination of mevalonic acid, mutational analysis and determination of enzyme activity should be performed in all cases with suggestive clinical symptoms and normal IgD levels.

## Genetic counseling and prenatal diagnosis

Genetic counseling should be offered to families at risk of MVA. As MVA is an autosomal recessive disorder, the risk for future pregnancies is 25% for families who already have a child with MVA.

Prenatal diagnosis of an affected fetus is possible by stable isotope-dilution gas chromatography/mass spectrometry, by determination of MVK activity in cultured amniocytes and biopsied chorionic villus [1,12,14,17], and by mutational analysis in informative families. In affected pregnancies, elevated levels of mevalonic acid have been detected in maternal urine [1,12,14]. Significant elevations of mevalonic acid have been detected in autopsied tissues (including lymph nodes, adrenals, ovaries, spleen, liver and brain) from an affected fetus, [14].

Prenatal diagnosis is usually not considered to be appropriate for HIDS.

## Management including treatment

There is no established therapeutic regime for patients with MVA. Dietary supplementation of cholesterol may reduce the frequency and severity of febrile attacks in some mildly affected patients, but may further compromise more severely affected patients [4]. An experimental trial with lovastatin in two patients with MVA resulted in clinical decompensation manifested by fever, acute myopathic changes, highly elevated creatine kinase activity, and worsened ataxia, diarrhea and vomiting. Intervention with corticosteroids (prednisone 2 mg/kg per day) was highly beneficial during clinical crises, with resolution of the crises within 24 hours. Additional long-term administration of ubiquinone-50, together with vitamin C and vitamin E, appeared to stabilize the clinical course and improve somatic and psychomotor development. The rationale is to correct the ubiquinone-50 deficiency and to increase the level of free radical scavengers [23].

Treatment of HIDS is difficult and largely supportive. Various standard anti-inflammatory drugs (including colchicine, non steroidal anti-inflammatory drugs (NSAIDs), steroids and thalidomide) have failed to suppress the attacks. Despite the severe side effects of 3'-hydroxy-3'-methylglutaryl-coenzyme A (HMG-CoA) reductase inhibitors in patients with classic MVA, a recently completed study showed a reduced excretion of mevalonic acid in all patients, a decreased number of febrile days in most of the patients with HIDS, and no side effects during simvastatin treatment [24]. Similarly, a beneficial effect of anakinra (a recombinant interleukin-1 receptor antagonist) was described in one patient [25].

## Prognosis

In early-onset multisystemic MVA, the prognosis is poor, with about half of patients succumbing in infancy or early childhood [4]. Mildly affected patients will develop short stature, progressive myopathy and, possibly, visual impairment due to uveitis, cataracts and tapetoretinal degeneration. The severity and frequency of the febrile attacks decline with age. In general, HIDS is considered to be a relatively benign condition. However, life expectancy may be reduced in some patients due to severe infections or the development of renal amyloidosis [26].

## Unresolved questions

It is still not known how the deficiency of MVK is related to the inflammatory periodic fever syndrome. Elevations of IgD, IgA and urinary leukotriene E4 excretion are probably secondary to stimulation of the immune system [27].

## Further information

: A comprehensive website for clinicians, scientists, patients and their carers. The site is maintained by the HIDS research group at the Department of General Internal Medicine, Radboud University Medical Center, Nijmegen, The Netherlands.

## Note

Figure 2 is reproduced with permission from Haas *et al*. 2001, © Georg Thieme Verlag KG
